# The use of dendrograms to describe the electrical activity of motoneurons underlying behaviors in leeches

**DOI:** 10.3389/fnint.2013.00069

**Published:** 2013-09-27

**Authors:** León J. Juárez-Hernández, Giacomo Bisson, Vincent Torre

**Affiliations:** Neuroscience Area, International School for Advanced Studies (ISAS-SISSA)Trieste, Italy

**Keywords:** leech, behavior, electrical activity, cross-covariance, dendrograms

## Abstract

The present manuscript aims at identifying patterns of electrical activity recorded from neurons of the leech nervous system, characterizing specific behaviors. When leeches are at rest, the electrical activity of neurons and motoneurons is poorly correlated. When leeches move their head and/or tail, in contrast, action potential (AP) firing becomes highly correlated. When the head or tail suckers detach, specific patterns of electrical activity are detected. During elongation and contraction the electrical activity of motoneurons in the Medial Anterior and Dorsal Posterior nerves increase, respectively, and several motoneurons are activated both during elongation and contraction. During crawling, swimming, and pseudo-swimming patterns of electrical activity are better described by the dendrograms of cross-correlations of motoneurons pairs. Dendrograms obtained from different animals exhibiting the same behavior are similar and by averaging these dendrograms we obtained a template underlying a given behavior. By using this template, the corresponding behavior is reliably identified from the recorded electrical activity. The analysis of dendrograms during different leech behavior reveals the fine orchestration of motoneurons firing specific to each stereotyped behavior. Therefore, dendrograms capture the subtle changes in the correlation pattern of neuronal networks when they become involved in different tasks or functions.

## Introduction

A major aim of Systems Neuroscience is the identification of patterns of electrical activity associated to sensory perceptions and underlying specific behaviors (Muller and Nicholls, 1974; Baader and Kristan, 1995; Reynolds et al., 1998; Nicholls, 2001; Kristan et al., 2005). These patterns of neuronal firing can be obtained with multi-unit electrodes (Takehara-Nishiuchi and McNaughton, 2008; Gullo et al., 2009; Luczak et al., 2013) and/or using imaging tools providing information on the global activation of brain areas (Grinvald and Hildesheim, 2004; Kerr and Denk, 2008; Wallace et al., 2008; Bonifazi et al., 2009; Vanni and Rosenström, 2011). The nervous system of most vertebrates is composed by millions and often billions of neurons and present experimental tools allow the recording of the electrical activity of a very small—and often negligible—fraction of these neurons and it is very difficult to relate patterns of electrical activity to specific behaviors. This issue is very challenging in vertebrates but can be better addressed in invertebrates, due to a lower number of neurons (Byrne et al., 1974; Stent et al., 1978; Kristan, 1982; Wittenberg and Kristan, 1992; Tsau et al., 1994; Frost and Kandel, 1995; Morris and Hooper, 1997).

In the invertebrate central nervous system of the leech, mechanical inputs to a segmental section of its body are transduced by 7 pairs of mechanosensory neurons; 3 specific for light pressure (touch or T cells), 2 for strong pressure (pressure or P cells), and 2 for noxious mechanical stimuli (N cells), (Kristan, 1982; Lewis and Kristan, 1998a,b; Pinato and Torre, 2000; Pinato et al., 2000; Arisi et al., 2001). The spontaneous behavior and the reaction to stimuli are mediated by 21 pairs of excitatory motoneurons and 7 pairs of inhibitory motoneurons, innervating the four different muscular synergies referred to as longitudinal, oblique, dorsoventral, and circular muscular fibers (Mason and Kristan, 1982; Norris and Calabrese, 1987; Lockery and Kristan, 1990a,b). These motoneurons have been extensively investigated using force and length transducers, imaging of muscle contractions and other electrophysiology tools (Stuart, 1970; Ort et al., 1974; Friesen et al., 1978; Kristan, 1982; Mason and Kristan, 1982; Norris and Calabrese, 1987; Zoccolan et al., 2001, 2002; Zoccolan and Torre, 2002; Garcia-Perez et al., 2004).

In the present manuscript we recorded and analyzed patterns of electrical activity from semi-intact leech preparations (Kristan et al., 1974a,b; Muller, 1981), where one or two ganglia of the mid body were exposed to allow long electrical recordings while the leech moved in the observation dish. Using up to 8 suction pipettes it was possible to record the electrical activity of some dozens of identified motoneurons. In order to characterize the patterns of their electrical activity, we used dendrograms describing in a compact way the hierarchy of correlations between motoneurons (Bialek and Rieke, 1992; Shadlen and Newsome, 1994, 1998; Fred and Jain, 2005; Kumar et al., 2010). These dendrograms capture the essence of the patterns of electrical activity underlying stereotyped behaviors and provide a good picture of the changes in the correlation structure among neurons during different behaviors (Contreras and Steriade, 1995; Bergman et al., 1998; Bair et al., 2001; Cohen and Maunsell, 2009; Jutras and Buffalo, 2010).

## Methods

**Figure 1:**
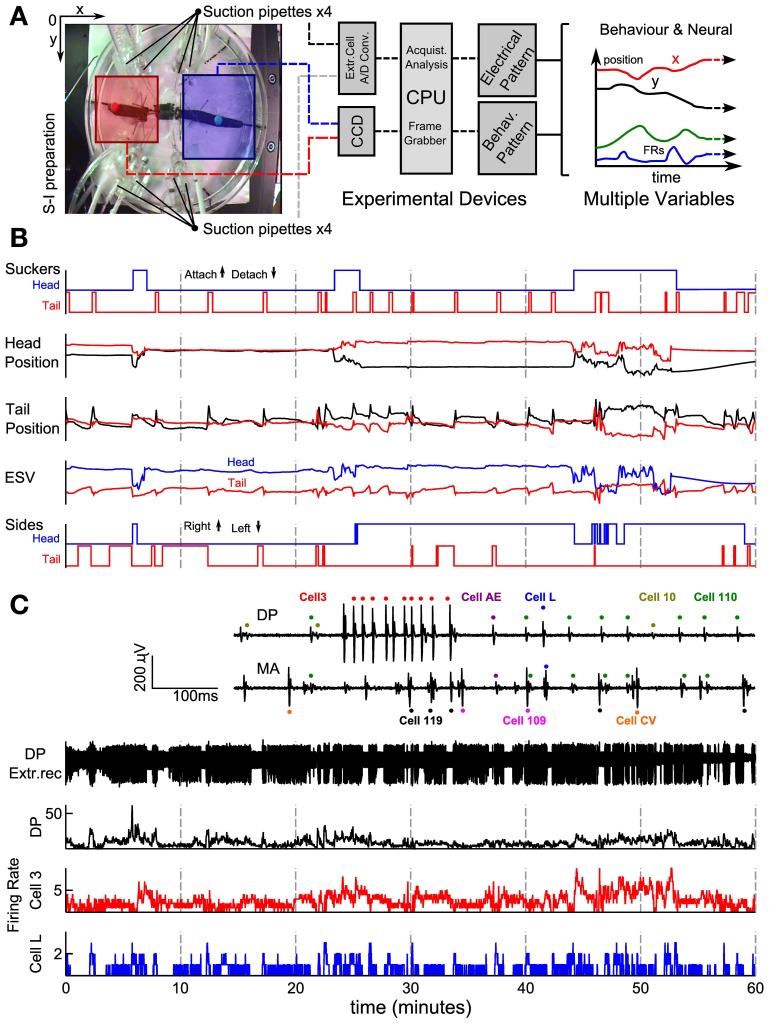
**Recording set-up and signal processing of behavioral and electrical data. (A)** Experimental set up showing an image of a semi-intact preparation taken with the CCD camera. Squares represent regions where the head (blue) and the tail (red) were usually located. An algorithm was able to detect and track the beads attached. Four suction pipettes were positioned at the left and four at the right side of the leech able to provide eight simultaneous electrical recordings. In the middle and left part of the panel a scheme of the overall system. **(B)** Behavior's characterization. Panel represents from top to bottom the time's evolution of sucker states, original head and tail coordinate positions (Cartesian coordinates x and y represented with red and black traces, respectively), ESV (Elongation-Shortening-Vector) and sides (steps) indicating whether the head and tail of the leech was at the right or left, respectively. Up indicates that the bead was located on the right side and down indicates that the bead was on the left side. **(C)** Electrical recordings. Inset shows extracellular recordings from the DP and MA nerves showing APs from motoneuron L (blue dots), 110 (green dots), AE (purple dots), 3 (red dots), 10 (yellow dots), CV (orange dots), Cell 109 (magenta dots), and 119 (black dots). From top to bottom: an example of an electrical recording from a DP nerve obtained with a suction electrode; firing rates of all APs recorded from that DP nerve (black), from an identified motoneuron 3 (red middle trace) and identified L (blue trace). AFRs binwidth 500 ms.

The set-up used in our experiments is illustrated in Figure 1A. Leeches were positioned on a Petri dish with a diameter of 15 cm (Figure 1A left). Eight suction electrodes were used to record extracellular APs from nerves and connective fibers. Two colored beads were glued over each leech's skin, next to its head and tail suckers. By using a CCD camera the position of the two colored beads was tracked in real time, and acquired data was transferred to a computer (Figure 1A middle). The leech behavior was monitored acquiring the *x, y* positions of the head (*x*, *y*)_*head*_ and tail (*x*, *y*)_*tail*_ (Figure 1A right). In this way it was possible to associate the behaviors (Figure 1B) to recorded patterns of electrical activity visually (Figure 1C).

### Animals and semi-intact preparation

Adult leeches *Hirudo species* obtained from Ricarimpex (Eysines, France) were kept at 5°C in tap water dechlorinated by previous aeration for 24 h. Before every experiment, animals were anesthetized with an 8% ethanol solution at room-temperature for 15–20 min. Leeches were extended and the skin was dried carefully. Beads of 4 mm diameter were glued on the dorsal side of leeches with Nexaband S/C tissue adhesive (Abott Labs, Chicago, USA) near the head and the tail. Once beads were correctly attached, leeches were moved to the Petri dish covered with sylgard elastomere (Corning corp., USA). The leeches were immersed in 150–200 ml chilled normal ringer solution (in mM: 115 NaCl, 1.8 CaCl_2_, and 4 KCl, 10 glucose and buffered with 10 Tris-maleate pH 7.4 with NaOH). Flaccid leeches (still under anesthesia) were pinned with entomological needles in the mid-body region. Individuals were dissected exposing one or two central ganglia (Figure 1A, right image). During the dissection, temperature was maintained at 6–8°C using a cold chamber. In some experiments, a complete skin segment (skin from mid dorsal to mid ventral) was left innerved from one side. At the end of the dissection, animals were left to recover from anesthesia and left to adapt to room temperature for 30 min. Experiments were performed at room's temperature (19–22°C) and semi-intact leeches were illuminated using a white light lamp without abrupt spatial and/or temporal gradients (Olympus Highlight 3100, Europe).

### Imaging

A color CCD camera (640 × 480 pixels; model WAT-231S; Watec, Tsuruoka, Japan) was used to acquire images of preparations (Figure 1A left image). Acquired images were sent to a frame grabber (PCI-1394; Texas Instruments) and then to a PC, able to process images in real time. Colored beads were tracked with a sample frequency of 25 Hz using an appropriate software program. Images were directly acquired in the hue/saturation/lightness color space. The detection of sucker attachments and detachments was obtained by visual inspection of videos recorded with the standard software VirtualDub (version 1.6.14; General Public License, 2006).

### Behavior's quantification off-line

In order to analyze and quantify the leech behaviors, several behavioral quantities (Figure 1B) were determined by using MATLAB (MathWorks, USA). The frequency of sucker attachment and detachment was determined from video recordings. An example from one experiment is visualized as two-step traces corresponding to the head (in blue) and tail (in red) sucker (Figure 1B *suckers*). Head and tail coordinates were recorded and represented as (*x*, *y*)_*head*_ and (*x*, *y*)_*tail*_ (Figure 1B: *Head* and *Tail positions*). The head and tail velocity (*v*_*x*_, *v*_*yi*_)_*head*_ and (*v*_*x*_, *v*_*y*_)_*tail*_ were calculated by convolution of the derivative with a Gaussian function as previously described (Mazzoni et al., 2005). A reference point was selected corresponding to the center of one of the two dissected ganglia: *M*(*x*, *y*). The head (tail) elongation was computed as the distance between (*x*, *y*)_*head*_ or (*x*, *y*)_*tail*_ and *M*(*x*, *y*). The total elongation of the leech was equal to the sum of the head and tail elongation.

Six different behaviors were previously detected in intact leeches (Garcia-Perez et al., 2005; Mazzoni et al., 2005). These stereotyped behaviors were: swimming, Exploring Head, Pseudo-swimming, Crawling, Peristalsis, and Stationary States; and could be detected also in semi-intact preparations and here were quantified. Brief description: during swimming, leeches detached both the head and tail suckers from the bottom of the dish and the head and tail beads detected oscillated with a frequency of ~1.5 Hz. In the exploring behavior leeches detached their head sucker keeping the tail sucker attached to the bottom and performed variable and irregular elongations/contractions without any periodicity. During pseudo-swimming (Mazzoni et al., 2005), leeches kept the tail sucker attached while the head oscillated as in swimming episodes. We considered swimming and pseudo-swimming similar behaviors as both consisted of a wave of rearward moving crests with similar frequencies (Kristan et al., 1974a,b, 2005). During crawling, leeches alternated sucker attachment and detachment elongating and shortening their bodies (Kristan et al., 2005). The coordinated activity of nine excitatory and inhibitory motoneurons of the longitudinal and circular muscles has been recorded in a previous work (Baader, 1997). The peristaltic behavior of leeches is characterized by the attachment of both suckers to the bottom of the recording dish and leeches elongate-contract their body irregularly with a frequency ~0.03 Hz. Leeches were in a stationary state when both of the suckers were attached to the bottom of the dish and leeches did not move.

### Electrical recordings

Suction pipettes were obtained from borosilicate glass electrodes (World precision instruments, Germany) pulled (P-97, Sutter Instruments, USA). The electrode tips were cut using a diamond microtome prism mounted over a manipulator under visual control through a stereoscopic microscope (Olympus SZ40, Europe). Electrodes with an inner diameter of ~200 μm were polished with a micro forge under visual inspection through an upright microscope (Zeiss, Germany).

Suction electrodes filled with normal ringer leech solution were connected to an extracellular recording amplifier (Pinato et al., 2000; Pinato and Torre, 2000; Zoccolan and Torre, 2002). Extracellular signals were digitized at 10 kHz by an A/D converter (model digidata-1322, 16 bit converter; Axon, molecular Devices, US) and data were transferred and stored on a PC computer. Signals were recorded and visualized using, respectively, Clampex v.8.1 and Clampfit v.9.2 software (Molecular Devices, USA). Electrical recordings were obtained from cleaned nerves or connectives (in *en passant* configuration) from one or two ganglia. Recordings from a single ganglion were obtained with 8 electrodes sucking the left and right of Anterior-Anterior (AA), Medial-Anterior (MA), Dorsal-Posterior (DP), and Posterior-Posterior nerves (PP) (Pinato et al., 2000; Arisi et al., 2001). Recordings from two ganglia were obtained with 8 electrodes sucking the left and right AA and DP nerves of both ganglia. Ganglia were dissected from the mid body between the 9th and the 13th segments.

For single ganglia recordings, glass capillaries (World precision instruments, Germany) were pulled (P-97, Sutter Instruments, USA) with a resistance of 18–20 MΩ when filled with potassium acetate solution (4 M). Electrodes were connected to the head stage of an amplifier (Axoclamp 2B, Axon Instruments, USA).

### AP detection

Action potential sorting was carried out by off-line analysis with MATLAB (MathWorks, Natick, USA) and specific APs shapes were identified, as shown in the inset of Figure 1C. Identification of motoneurons was cleared by comparing extracellular signals, intracellular recordings from visually identified motoneurons and bibliographic data (Ort et al., 1974; Pinato and Torre, 2000; Zoccolan and Torre, 2002). As example, APs elicited by motoneurons 3 and 107 produce the largest extracellular signals observed on the DP and AA nerve, respectively. Extracellular APs of motoneurons annulus erector (AE) and longitudinal (L) were identified because they were visible in both MA and DP root nerves. Other cells were identified (inset Figure 1C).

### Average firing rate and cross-covariance coefficient

The duration of the recording was divided into bins of constant width. Different widths were used to compare properties at different time scales (varying between 100 and 500 ms). For each single neuron the number of APs occurring in each bin was counted and the resulting discrete time series represented the neuron average firing rate (AFR). The same procedure was applied to nerves providing the nerve AFR. For pairs of neuron AFR—or pairs of nerve AFR—the time-varying unbiased cross-covariance of the neuron (nerve) firing rates was computed in a bin of 50 s as:

(1) $$\rho_{12}(m)\{\begin{array}{ll} \frac{1}{N-|m|}\sum_{n=0}^{N-|m|-1}\left[f_{1}(n+m)-\frac{1}{N}\sum_{i=0}^{N-1}f_{1}(i)\right] & \\ \;\left[f_{2}(n)-\frac{1}{N}\sum_{i=0}^{N-1}f_{2}(i)\right], & m\geq 0\\ \rho_{21}(-m), & m<0 \end{array}$$

where *f*_1_ and *f*_2_ represent the pair of firing rates. *N* is the numbers of samples (commonly set to 100). The sampled window was chosen in order to ensure a reliable estimate of the covariance coefficients at low lags. The obtained value ρ_12_(0) was assumed to be located in the point corresponding to the center of the time window. A single, scalar value, was then obtained by averaging ρ_12_(*m*) for *m* in the range [−5, 5]. This procedure reduced noise around 0, where the cross-covariance is usually large.

### Dendrograms

The firing rates of up to 30 APs sorted motoneurons were classified into clusters, with similar properties. One of the most intuitive measures of similarity between two firing rates is the Pearson's correlation coefficient, providing an M×M matrix when the firing of *M* neurons is available. In our case, we want to investigate how single neuron activities are related to each other and if the M×M matrix of cross-covariance can be clustered in a suitable hierarchy providing a compact coding of electrical patterns underlying a stereotyped behavior, such as swimming or crawling: this leads to hierarchically-nested sets of partitions, commonly represented in a nerved tree diagram, or dendrogram (Beggs and Plenz, 2004). In our study we used the Person distance (i.e., one minus the sample Pearson's correlation between single cell activities) as a metric and a dissimilarity matrix was constructed using the functions pdist and squareform implemented in MATLAB. The dissimilarity matrix [*d*_*ij*_] contains the M×M pairwise dissimilarity values:

(2) $$d_{ij}=1-\frac{\begin{array}{l} \sum_{n=0}^{N-1}\left[f_{1}(n)-\frac{1}{N}\sum_{i=0}^{N-1}f_{1}(i)\right]\\ \;\left[f_{2}(n)-\frac{1}{N}\sum_{j=0}^{N-1}f_{2}(i)\right] \end{array}}{\sqrt{\begin{array}{l} {\sum_{n=0}^{N-1}\left[f_{1}(n)-\frac{1}{N}\sum_{i=0}^{N-1}f_{1}(i)\right]}^{2}\\ \sum_{n=0}^{N-1}{\left[f_{2}(n)-\frac{1}{N}\sum_{j=0}^{N-1}f_{2}(j)\right]}^{2} \end{array}}}$$

where *f*_1_ and *f*_2_ represent the pair of firing rates and *N* the sample number. Once the dissimilarity matrix was obtained, the hierarchical cluster tree was obtained by means of the linkage function (using the complete linkage method) and visually represented with the dendrogram function, both implemented in MATLAB. We used the complete linkage method because it is less sensitive to noise (Baker, 1974) and may be used with non-Euclidean dissimilarity matrices, such as the uncentered correlation. The hierarchical clustering procedure explained above was run on time intervals during which the leech was either swimming, pseudo-swimming, crawling, elongating, or shortening. Separate episodes were grouped together.

#### Comparison among dendrograms

By considering time intervals during which the animal was crawling, swimming, or pseudo-swimming, as well as shortening or elongating, we estimated a dendrogram for each of these behaviors, by running the aforementioned hierarchical clustering algorithm of sorted APs from up to 30 motoneurons. Once these dendrograms were estimated, we used them as templates to be compared with a generic dendrogram calculated on a sliding time-window of 100 s width and 2 s sliding step. To compare two dendrograms, one needs a measure of similarity between them. Usually, similarity indexes are normalized and range between zero and one, where 0 represented complete dissimilarity and 1, complete equality. We obtained the best comparisons both with Fowlkes-Mallows Index (*B*_*k*_) and Normalized Mutual Information (Fred and Jain, 2005). Let be

- *n*_11_ the number of pairs of elements that are in the same cluster in both dendrograms.
- *n*_00_ the number of pairs of elements that are in different clusters in both dendrograms.
- *n*_10_ the number of pairs of elements that are in the same cluster in the first dendrogram, but in different ones in the second dendrogram.
- *n*_01_ the number of pairs of elements that are in different clusters in the first dendrogram, but in the same cluster in the second dendrogram.

then, Fowlkes-Mallows Index (*B*_*k*_) is defined as the geometric mean between the ratio *n*_11_/(*n*_11_ + *n*_10_) and the ratio *n*_11_/*n*(*n*_11_ + *n*_01_). In order to apply the information theory to clustering, we need to introduce the definition of entropy: assuming that all *n* elements that belong to the set *X* have the same probability of being picked and choosing an element of *X* at random, the probability that this element is in cluster *C*_*i*_ ∈ *C* (where *C* is the set of clusters) is:

$$P(i)=\frac{\left|C_{i}\right|}{n}$$

where |*C*_*i*_| is the number of elements that belong to cluster *C*_*i*_. Then, the entropy associated with a clustering *C* is:

$$H(C)=-\sum_{i}P(i)\log_{2}P(i)$$

Informally, the entropy of a clustering *C* is a measure for the uncertainty about the cluster of a randomly picked element. The notion of entropy can be extended to that of mutual information, which describes how much we can on the average reduce the uncertainty about the cluster of a random element when knowing its cluster in another clustering of the same set of elements. Formally, the mutual information between two clusterings *C*, *C*′ is defined as:

$$I(C,C^{\prime })=\sum_{i}\sum_{j}P(i,j)\log_{2}\frac{P(i,j)}{P(i)P(j)}$$

where *P*(*i*, *j*) is the probability that an element belongs to cluster *C*_*i*_ in *C* and to cluster *C*_*j*_ in *C*′:

$$P(i,j)=\frac{\left|C_{i}\cap {C^{\prime }}_{j}\right|}{n}$$

The mutual information *I* is a metric on the space of all clusterings. However, it is not bounded by a constant value which makes it difficult to interpret. As the mutual information between two clusterings is bounded by their entropies

$$I(C,C^{\prime }\leq \min\{H(C),H[{\left(C\right]}^{\prime })\}$$

a normalization by the geometric or arithmetic mean of the entropies seems to be reasonable. A different normalization was proposed by Fred and Jain (2005) as:

$$NMI(C,C^{\prime })=\frac{2I(C,C^{\prime })}{H(C)+H(C^{\prime })}$$

For which we have:

$$0\leq NMI(C,C^{\prime })\leq 1$$

**Figure 2:**
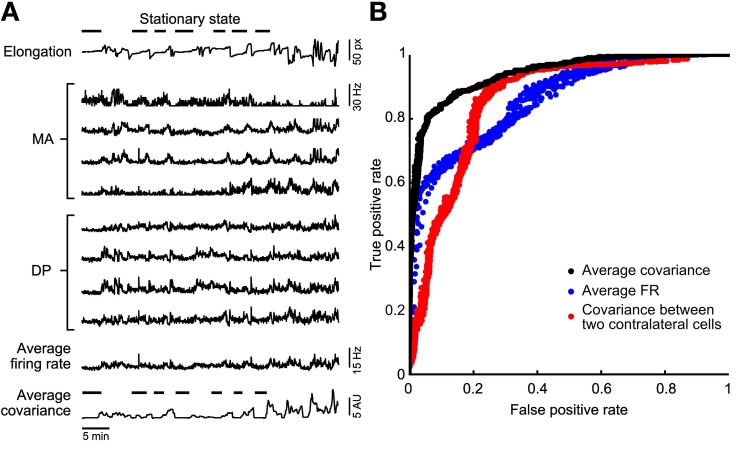
**Stationary states classification. (A)** A representative experiment in which the leech elongation was measured (top trace, with a discontinued line emphasizing the lack of motion during identified stationary states) and compared to the firing rate recorded from four DP and four MA nerves located in the 11th and 12th segmental ganglia (mid traces). Almost bottom: reproduction of the the *Average Firing Rate* (<AFR_i_>) obtained from the eight firing rates averaged. Bottom trace: *Average Covariance* (<ρ_*ij*_>) obtained from the average of all entries of correlations between firing rates. **(B)** Comparison of the different classifiers using a ROC analysis. A variation of the binarization parameters (see methods) provides a ROC curve. The perfect classifier is represented by the point (0, 1). Three classifiers are compared: the binarized <ρ_*ij*_> (black dots), the <AFR_i_> (blue dots), and the binarized cross-covariance calculated using only two contra lateral motoneurons 3. The binarized <ρ_*ij*_> classifier represents the best choice as its curve is the closest to the perfect classifier point.

These similarity indexes rely on the integer parameter *k* that represents the threshold at which both dendrograms are cut for the comparison, so that only *k* clusters are selected (Fowlkes and Mallows, 1983). In our analyses we always considered *k* = 2 for crawling dendrograms, and *k* = 6 for swimming and pseudo-swimming. This choice was determined in order to maximize the yield of our classification. Values near to 0 are observed comparing a dendrogram obtained when the animal is stationary (see Figure 2). Average dendrograms for crawling, swimming, and pseudo-swimming were estimated from the average activities of the 6 more reliable recorded motoneurons, i.e., cells CV, L, 3, 109, 102, and 119. The *averaged dendrogram* <*D*_*behavior*_> for crawling, swimming, and pseudo-swimming, respectively, was estimated using all the recorded episodes in 5 experiments (for each of the behaviors) and using the AFRs of the 6 most reliably identified cells. Similarity indexes between the estimated dendrograms in each behavioral episode and the average dendrograms (*D*, <*D*_*behavior*_>) were then calculated and compared with the average over similarity indexes between all average dendrogram couples <s(*D*, *D*′)>.

### Discriminant analysis

Another approach to decode the behavioral state of the leech from sorted APs, is to run a discriminant analysis on time-varying features believed to characterize the behavior, such as the *n*(*n* − 1)/2 coefficients of the covariance matrix estimated. We used the function classify implemented in MATLAB, and passing, as input argument, the matrix of all the time-varying covariance coefficients and a short training episode, half containing 0-class events (i.e., not during the behavior under consideration) and half containing 1-class events (i.e., during the behavior under consideration). We tested different types of classifier options: linear, which fits a multivariate normal density to each group, with a pooled estimate of covariance, diaglinear, i.e., naive Bayes classifier with a pooled estimate of covariance, quadratic, which fits multivariate normal densities with covariance estimates stratified by group and diagquadratic, i.e., naive Bayes classifier with covariance estimates stratified by group. The function output function has a unit value when the behavior under consideration is performed and 0 otherwise. When testing these classifiers on all the 276 covariance matrix coefficients, we reduced the dimensionality. This was achieved with a principal component analysis on the original covariance matrix (pcacov function, MATLAB), and selecting the first 100 principal components.

### ROC analysis

To assess the performance of a classifier (the threshold classifier, the discriminant analysis or the dendrogram matcher) we used Receiver Operating Characteristics (ROC) graphs (Gabbiani and Cox, 2010). We used the <AFR_i_> and <ρ_*ij*_> as threshold classifiers (see Figure 2B), where the triangular bracket notation indicate average over index *i* (for AFR) and average over indexes *i* and *j* (for ρ). Given a classifier and an instance, there are four possible outcomes. If the instance is positive and it is classified as positive (e.g., high values of <AFR_i_> or <ρ_*ij*_>), it is counted as a true positive; if it is classified as negative, it is counted as a false negative. If the instance is negative and it is classified as negative, it is counted as a true negative; if it is classified as positive, it is counted as a false positive. The true positive rate (TPR) of a classifier is estimated as the ratio of the true positives count to the sum of the true positives and false negatives counts; the false positive rate (FPR) of a classifier is estimated as the ratio of the false positives count to the sum of the false positives and true negatives counts. ROC graphs are two-dimensional graphs in which TPR is plotted on the Y axis and FPR is plotted on the X axis. The perfect classifier is represented by the point (0, 1). By varying the classifier type (as in the discriminant analysis case) or the threshold parameter (Figure 2B) different performances are obtained. One classifier in the ROC space is better than another if TPR is higher; FPR is lower, or both. We investigated the ROC space and determined which classifier performed better. For threshold classifiers obtained in Figure 2B, signals were processed using the <AFR_i_> or <ρ_*ij*_> values.

We set a variable threshold *T* and for each value of *T* we identified all times *t*_<AFRi>_ for which <AFR_i_> was less than *T* and all times *t*_<ρ*ij*>_ for which <ρ_*ij*_> was less than *T*. For each value of *T* we computed the rate of true positive identifications, i.e., all the times properly identified as a stationary state and the rate of false positive identifications, i.e., all the times erroneously identified as a stationary state. These true and false positive identifications were computed for both *t*_<AFR_*i*__> and *t*_<ρ*ij*>_ as a function of *T* providing a pair of numbers corresponding to the true and FPRs.

## Results

Semi-intact leeches can be studied for several hours while their electrical activity is recorded and their behavior monitored allowing the identification and characterization of patterns of electrical activity underlying specific behaviors (Figure 1). Indeed, they could exhibit their stereotyped behaviors: crawling (Figure 5), whole body shortening or elongation (Figure 6) or perform swimming or pseudo-swimming (Figure 7). In a typical experiment, after dissection, we obtained variables originated from a coordinate system; the most important was the elongation vector: the distance between beads glued to their body extremes (Figure 1A; see also methods). A sudden decrease of the elongation indicated a contraction and an increase of it: elongation. Variables and video recordings analyzed together permitted us to precise the performing of behaviors.

### Electrical patterns underlying stationary states

Intact leeches as well as semi-intact leeches spent a considerable time in stationary states, during which they do not move and when the head and tail velocities were negligible and both suckers were attached to the bottom of the recording dish. Leeches were assumed to be in a stationary state when the head *v*_head_ and tail velocity *v*_tail_ were less than 3σ (standard deviation) for longer than 10 s, typically resulted in σ2.5 pix/s.

From the analysis of the leech elongation we identified the stationary states and indicated them by the horizontal bars over the upper trace in Figure 2A. During these identified behaviors, we compared the averaged firing rate <AFR_i_> from all the electrical signals recorded: 4 MA and 4 DP root nerves. We measured also the covariance ρ_*ij*_ between the firing of APs recorded from all pairs of the nerves mentioned. In order to have a global measurement of the correlation of the overall electrical activity we averaged ρ_*ij*_ over all pairs of nerves, so to obtain the average covariance <ρ_*ij*_>. As shown at the bottom of Figure 2A, during stationary states both <AFR_i_> and <ρ_*ij*_> had low values. Therefore, we asked which features of the electrical activity characterize stationary states and whether stationary states are better identified by a low value of <AFR_i_> or of <ρ_*ij*_>.

In order to understand which features best identify stationary states we constructed a ROC curve (Figure 2B). We set a variable threshold *T* and for each value of *T* we identified all times *t*_<AFRi_> for which <AFR_*i*_> was less than *T* and all times *t*_<ρ*ij*>_ for which <ρ_*ij*_> was less than *T*. The value of *T* for <AFR_i_> varied from 1 to 20 Hz and for <ρ_*ij*_> varied from 0.01 to 1 Hz^2^. These pairs are plotted in Figure 2B, for the classifier based on <AFR_i_> (blue dots) and <ρ_*ij*_> (black dots), respectively. When the value of *T* decreased, the rate of both true and false positives decreased. As shown in Figure 2B, the identification of the stationary state based on the average cross-covariance <ρ_*ij*_> approaches the ideal classifier better than the identification based on average firing rates <AFR_i_>. The best accuracy for the firing rate-based classificator is reached for a threshold that ranges between 2.5 and 4 Hz and for the correlation-based classificatory is reached for a threshold that ranges between 0.1 and 0.3 Hz^2^. Identification of stationary states based on the cross-covariance ρ_*ij*_ of a single pair of identified contra lateral motoneurons (red dots) is less efficient than the identification based on properties averaged over a population of neurons. We repeated the same analysis with 15 leeches and in 13 of them we found that stationary states were better identified with the average cross-covariance <ρ_*ij*_>. In the remaining two cases, stationary states could be equally well-identified using <ρ_*ij*_> and <AFR_i_>.

### Electrical patterns underlying the sucker attachment and detachment

We investigated the statistical properties of sucker attachment and detachment (Muller and Nicholls, 1974; Baader and Kristan, 1995; Reynolds et al., 1998; Kristan et al., 2005).

**Figure 3:**
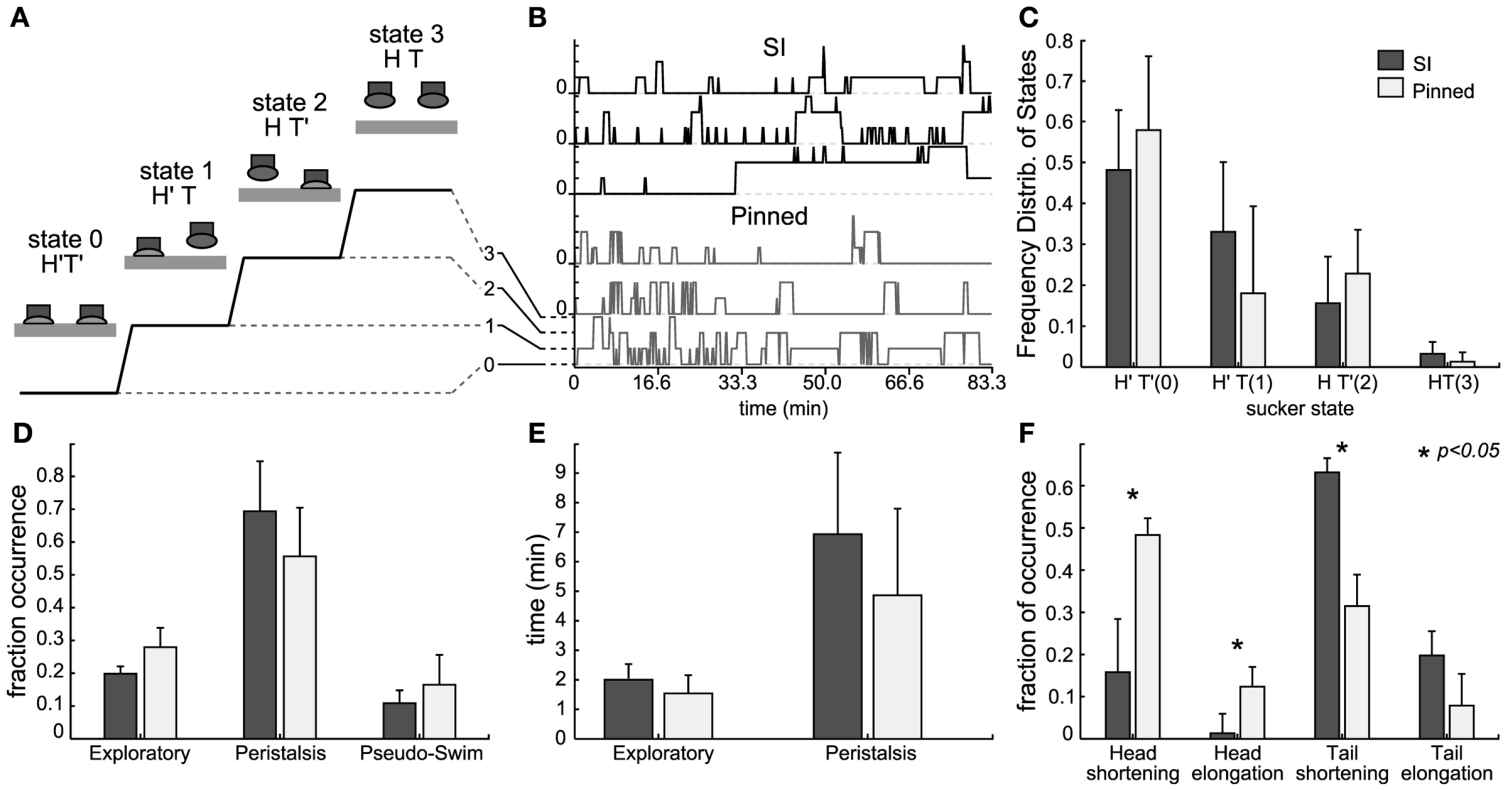
**Behaviors from head and tail sucker. (A)** Diagram representing the four behavioral states of the suckers: in state 0, both suckers are attached (*H*′*T*″), in state 1, the head sucker is attached while the tail sucker is detached (*H*′*T*), in state 2, the head sucker is detached while the tail sucker is attached (*HT*′), in state 3 the head and tail suckers are both detached (HT). **(B)** Example of sucker state evolution from three semi-intact experiments (top three traces in black) and three pinned experiments (bottom three traces in gray). **(C)** Frequency distribution of the sucker states averaged from 12 experiments. Data from semi-intact leeches in dark gray and from pinned-intact in light gray. **(D)** Fraction of occurrence of exploratory, peristalsis, and pseudo-swimming when the tail sucker was attached but the head sucker was not (state *HT*′). Comparison between pinned but intact (black bars) and semi intact (white bars) leeches. **(E)** Mean duration (minutes) of exploratory and peristalsis episodes averaged from experiments with 10 pinned-intact (white bars) and 12 semi-intact (black bars) leeches. **(F)** Fraction of occurrence of body contraction and elongation following head and tail detachment of pinned but intact (black bars) and semi intact (white bars) leeches.

We analyzed and compared the statistics of suckers attachment and detachment in intact pinned leeches (mid body) and semi-intact leeches (Figure 3). The head sucker could be attached (*H*′) or detached (*H*) and similarly tail sucker could be attached (*T*′) or detached (*T*). Leeches could enter into four different states: *H*′*T*′ (state 0), *H*′*T* (state 1), *HT*′ (state 2), and *HT* (state 3). Transitions between these states were represented over the time (see Figure 3A). Both preparations attached and detached their suckers spontaneously and we quantified the sucker states in both preparations (*n* = 4 each case) recorded for several hours (Figure 3B). As shown in Figure 3C, for semi-intact leeches the fraction of time spent in states 0–3 was, respectively, 0.48 ± 0.20, 0.33 ± 0.15, 0.16 ± 0.11, and 0.03 ± 0.03; and for intact-pinned leeches the fraction of time spent was 0.58 ± 0.29, 0.18 ± 0.20, 0.23 ± 0.12, and 0.01 ± 0.02, respectively. State 0 was predominant while the state 3 almost never occurred in both preparations. Peristalsis-like behavior was observed when leeches presented short combined contractions and elongations and suckers stayed in state 0 (similar to Mazzoni et al., 2005). When leeches stayed in state 2, their tail sucker was attached and their head could move producing different behaviors. Indeed leeches could explore, stretch or relax their head or perform pseudo-swimming (Figure 3D). These behaviors were not significantly different between semi-intact and intact-pinned leeches (Figure 3E). An intact pinned leech shortened its head to mid-body segment more often than a semi-intact leech (Figure 3F) but the semi-intact leech shortened its tail to mid-body segments more often than an intact pinned leech (Figure 3F). The results shown in Figure 3 suggest that semi-intact leeches are able to attach and detach their head and tail suckers similarly to what observed in intact-pinned leeches.

We next asked whether we could identify patterns of electrical activity underlying sucker attachment and detachment.

Having identified transitions between different sucker states, we aligned the electrical activity recorded during these transitions and investigated events in a time window of 30 s before and 30 s after sucker attachment or detachment. In these experiments (*n* = 15), we used suction electrodes (*en passant* configuration) to record the electrical activity also from the connectives (single ganglion). One electrode was situated in the rostral side (RC) of the ganglion and another electrode in the caudal side (CC). Other electrodes were used to record from the DP and MA nerves.

**Figure 4:**
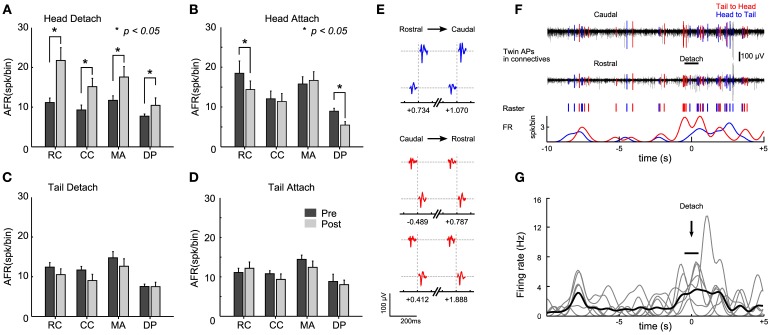
**Identification of electrical patterns underlying head sucker detachment. (A)** Averaged <AFR_*j*_> over 30 s before (black bars) and after (gray bars) head sucker detachment. *AFRs* obtained from 12 semi-intact leeches in which the electrical activity of the rostral connective (RC), caudal connective (CC), medial anterior nerve (MA), and dorsal posterior nerve (DP) were recorded. **(B–D)** As in **(A)** but for the head attachment, tail detachment, and tail attachment, respectively. **(E)**. Twin APs identified from connective recordings during the head detachment shown in **(F)**. Three pairs of APs with a constant delay ~6 ms were identified. Twin APs in blue correspond to APs traveling from head to tail (and in red for those traveling from tail to head). The number below each twin indicates the occurrence time between the couple before (negative value) or after (positive value) the detach (time, 0 ms). **(F)** Isolated twin spikes during head sucker detachment. Top trace: electrical recordings from the two connectives (black). APs with a fixed latency of ~6 ms were isolated and highlighted in blue (head to tail) and red (tail to head). Middle trace: raster plot from twin APs. Bottom trace: smoothed AFR from twin's APs (bin: 1 s). Notice the raise in the AFR at the time of detachment (black bar). **(G)** Smoothed AFR of 7 twin units (gray traces) isolated from different experiments: previous bursts coincided ~8 s before head detachment; <AFR_units_> in black. Detachment events (marked with the arrow) were determined with an uncertainty of about 1 s (indicated by the horizontal bar). ^*^Student's T-test, *p* < 0.05.

When the head sucker detached and the leech shortened its whole body, we observed that the AFR increased by more than 2 times in the connectives and roots nerves (Figure 4A, Student's *T*-test, *p* < 0.05). When the head sucker attached, the AFR decreased by 50% in the RC and in the DP nerves, but not in the CC and in the MA nerves (Figure 4B). We could not detect any significant change (Student's *T*-test, *p* < 0.05) in the AFR from connectives and root nerves either when the tail sucker detached (Figure 4C) or attached (Figure 4D). As we were able to detect a significant increase of the AFR of signals measured from the connective fibers during the head sucker attachment, we analyzed the APs traveling along the connectives with the aim to identify possible command signals for this behavior. We observed (*n* = 7) twin APs in CC and RC separated by a delay of 4–6 ms (Figure 4E) that could travel from head to tail and *vice versa* (respectively, blue and red in an experiment, Figure 4F). Twin APs increased their AFR around 8 s before the detachment and then a larger burst of APs appeared during sucker detachment (time 0 in Figure 4F). This pattern was found in 85% of head sucker detachments and APs traveling from the tail to the head appeared consistently (Figure 4G, Student's *T*-test, *p* < 0.05).

### Electrical patterns underlying crawling

Previously, we found that during stationary states both <AFR_i_> and <ρ_*ij*_> decreased and became almost negligible. In contrast, when the leech moved, we observed large values of <AFR_i_> and <ρ_*ij*_>. Therefore, we asked whether we could infer from the firing rate of motoneurons if an individual performed elongation or contraction of its body or even if it could perform swimming, pseudo-swimming or crawling.

**Figure 5:**
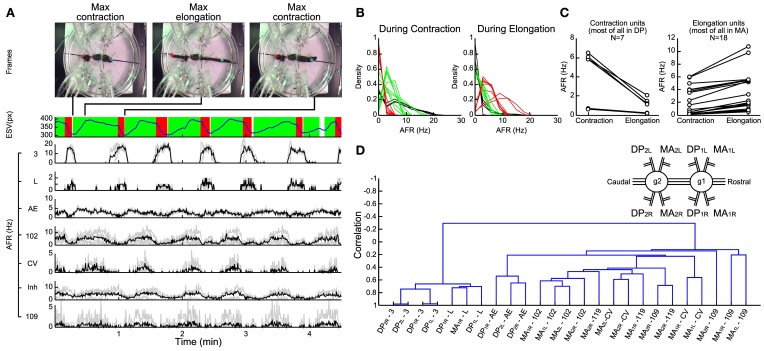
**Electrical patterns underlying crawling. (A)** Frames from a crawling episode performed by the leech: whole body contraction (first frame), elongation (second frame), and a new contraction (third frame) which corresponded to the temporal performing elongation signal (blue trace below). The contraction phase (red) and the elongation phase (green) were defined by processing the first time-derivative of the elongation. A custom supervised AP-sorting algorithm was run on the 8 available electrical recordings (4 DPs and 4 MAs). Twenty-five different units were detected, classified, and presented (3, L, AE, 102, CV, and 109). The AFR from other unidentified inhibitors was averaged (indicated as Inh). **(B)** Density plots of the average firing rate of each unit during contraction (left plot) and elongation phases (right plot). Three families of neurons were distinguished: pure contractors (black traces), pure elongators (red traces), and intermediate units (green traces). **(C)** Scatter plots of the average firing rate of each unit detected in the DP nerve (left plot) and in the MA nerve (right plot). During elongation, most of the units identified in the DP nerve decrease their *AFR*, while the opposite happened in MA units. **(D)** Dendrogram obtained from a hierarchical clustering, using the single-linkage method with the correlation as a metric. The resulting dendrogram returned two families of neurons, the activities of which are anti-correlated: the contractor neurons are listed on the left, while the elongators are on the right. Nerve names are reported in the inset; i.e., DP_2L_, Left Dorsal Posterior nerve of the 2nd ganglion (1, rostral; 2, caudal). Ganglia are numbered for simplicity: numberings are not referred to actual segments.

Pinned (*n* = 8) and semi-intact leeches obtained with limited harm (*n* = 7) displayed stereotyped behaviors such as crawling and swimming. A crawling phase is shown in the upper frames of Figure 5A. In this behavior the elongation vector (blue trace in Figure 5A) oscillated with a period of ~40 s. During the contraction phase (highlighted in red; Figure 5A) several longitudinal excitatory motoneurons, such as 3 and L increased their AFR (top of AFR in Figure 5A) while others cells such as AE, CV, 102,109 (Figure 5A) decreased their AFR. The AFR of these motoneurons was inverted in elongation phases (highlighted green; Figure 5A).

In several experiments (*n* = 14) we identified the APs from different motoneurons and their AFR during contraction (left panel of Figure 5B) and elongation (right panel of Figure 5B) performed in a crawling event. From this analysis we identified three classes: pure *contractors* activated only during the contraction phase (black traces, comprising 3 and L), pure *elongators* activated primarily during the elongation phase (red traces comprising 102 and CV) and intermediate units activated both during contraction and elongation (green traces). In agreement with previous observations (Stuart, 1970; Kristan et al., 1974b; Ort et al., 1974; Baader and Kristan, 1992), most of the pure contractors were found in the DP nerve and most of the pure elongators in the MA nerve (Figure 5C).

In order to identify and characterize patterns of electrical activity during crawling we developed a hierarchical clustering of motoneurons using the single-linkage method with the correlation as a metric (see Methods). This procedure returned a dendrogram describing in a compact form the degree of correlated and anti-correlated activity of motoneurons during crawling (Figure 5D). Interestingly this analysis identified two groups correlated among them, respectively, during the contraction and elongation phases. The resulted branches denoted that both groups are mutually anti-correlated: the AFR of one increases during contraction and the other during elongation. The dendrogram shows that dorsal exciters 3 and L from neighbor ganglia and contra lateral ones were highly correlated among them (left arm, Figure 5D) forming a solid arm, while other excitors, such AE and 102 were similarly correlated to each other (but to a lesser extent) to the left and belonged to the arm in the opposite position. At the opposite side of the dendrogram, other groups of motoneurons were formed (right arm: two CV cells and four 109) and they were anti-correlated to motoneurons of the left arm of the dendrogram. These observations were analogous in other experiments (*n* = 7) where semi-intact leeches exhibited a clear and recognizable crawling.

### Electrical patterns underlying contraction and elongation

**Figure 6:**
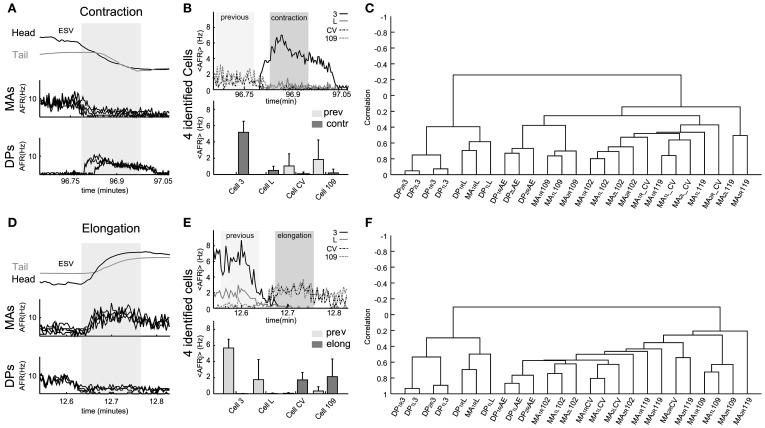
**Electrical patterns underlying contraction and elongation.** Examples of time varying *AFR* from nerves and identified neurons during contraction (respectively, **A** and **B**) and elongation (respectively, **D** and **E**) obtained from an experiment used to calculate the corresponding dendrogram **(C,F)**. **(A)** Response of eight nerves (4 DP and 4 MA) to the whole body contraction elicited during 30 s (dark zone, scale in minutes) of an experiment. The 4 MA and 4 DP nerves exhibited, respectively, decay and rise in their *AFR* during contraction. **(B)** Top: traces show the <AFR_*j*_> from 4 cells of the same type (e.g., four cells 3). Identified motoneurons were cell 3, cell L, cell CV, and cell 109. Bottom bars: mean of the <AFR_*j*_> in 30 s episodes of previous (light zone) and contraction (dark zone) calculated in each cell. Excitors from DP exhibited a rise in their activity (in Hz): Cell 3 rose from 0 to 5.72 ± 1.3; cell L, from 0 to 0.71 ± 0.35. Inhibitors exhibited a fall in their activity: cell CV, from 1.02 ± 1.3 to 0.18 ± 0.15; and, cell 109, from 1.88 ± 1.9 to 0.20 ± 0.15. **(C)** Dendrogram obtained from the hierarchical analysis computed from all the contraction episodes detected during the whole experiment. **(D)** Similar plot as in **(A)** for an elongation lasting about 30 s (dark zone). **(E)** As in **(B)**, top traces show the <AFR_*j*_> from the same cells during elongation. Excitors from DP decreased their activity: Cell 3, from 5.89 ± 0.99 to 0; cell L, from 1.61 ± 1.85. Inhibitors exhibited a fall in their activity: cell CV, from 0.01 to 1.56 ± 0.6; and, cell 109, from 0.32 ± 0.2 to 2.12 ± 2.30. **(F)** Dendrogram obtained from the hierarchical analysis from all elongation episodes detected during the whole experiment. Names of the nerves as in Figure 5.

We analyzed the electrical activity of motoneurons during contractions and elongations not associated to crawling to know if there could be variation with dendrograms originated from crawling episodes (Figure 6).

During all contraction episodes, the AFR recorded from the DP nerves increased and from the MA nerves decreased (Figure 6A). The AFR of motoneurons 3 and 109 increased during contractions and the AFR of CV and 109 decreased (Figure 6B). An almost opposite behavior was observed during elongations (Figures 6D,E): during these events the AFR from the MA nerves increased around 2–5 times as well as the AFR of motoneurons CV and 109 and the AFR from the DP nerves and of longitudinal excitatory motoneurons, such as 3 and 5 decreased to values close to 0.

Electrical patterns of motoneurons firing during all elongations—either during crawling or during exploration—were similar, with a pattern almost opposite to the one observed during contractions. However, the dendrograms during contraction (Figure 6C), elongation (Figure 6F) and during crawling were similar but not identical. The three dendrograms have two major arms of anti-correlated activity, with each arm composed of mutually correlated motoneurons. However, the degree of correlated activity varied with a specific behavior: motoneurons 3 and L were more correlated during the contraction phase of crawling than when the leech contracted its body during other behaviors (compare Figures 5D with 6C or 6F). These observations were repeated and averaged in 7 experiments where semi-intact leeches crawled and explored the environment contracting and elongating their whole body (behavioral statistics not shown).

### Electrical patterns underlying swimming and pseudo-swimming

**Figure 7:**
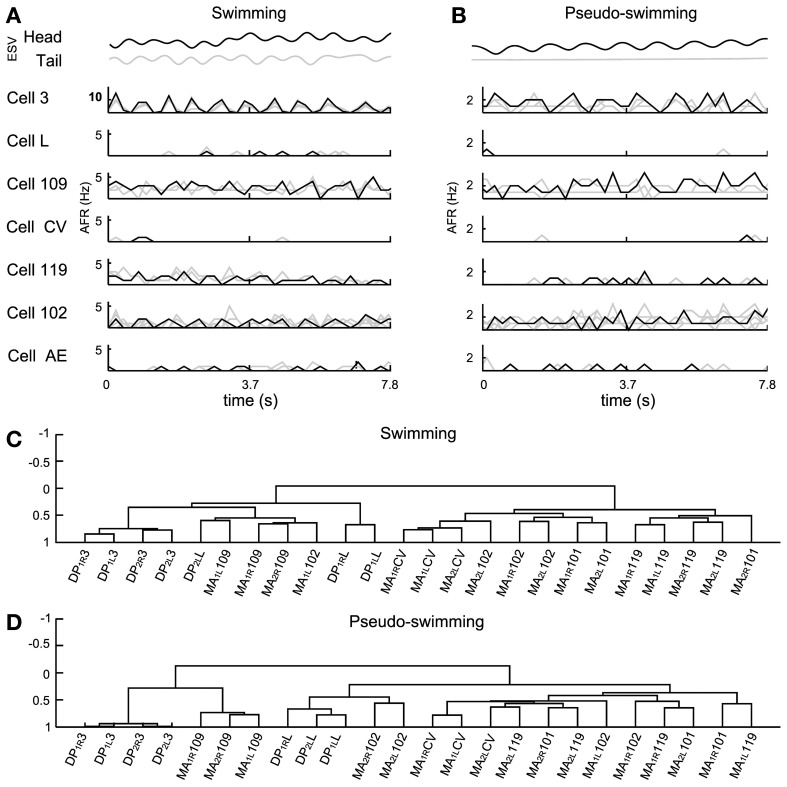
**Electrical patterns underlying swimming and pseudo-swimming.** Episodes of swimming and pseudo-swimming obtained from a semi intact preparation which coincided in duration (7.8 s). **(A)** Swimming episode. From top to bottom: head and tail elongation (*ESV* in black and gray, respectively) and the firing rate from motoneurons 3, L, 109, CV, 119, 102, and AE. Extracellular recordings from four MAs and four DPs allowed the identification of four motoneurons for each type (gray traces) which were also averaged (AFR: black traces). **(B)** Elongation and firing rates as in panel **(A)**, but for the pseudo-swimming behavior (see the smooth trace of ESV_tail_ in gray). **(C,D)** Dendrograms obtained from the hierarchical analysis during the swimming and pseudo-swimming episodes respectively. In both hierarchical analysis the AE motoneuron was excluded. Ordinate axis in both dendrograms shows the level of correlation. Names of the nerves as in Figure 5.

As was expected, swimming and pseudo-swimming could also be observed in semi-intact leeches. Indeed, was possible to observe the head and tail oscillating with a frequency close to 1 Hz (Figure 7A).

Often, the head of semi-intact leeches oscillated with frequencies varying from 0.5 to 2 Hz, while the tail remained with its sucker firmly attached to the bottom of the recording dish (Figure 7B). These episodes were identified as pseudo-swimming (Garcia-Perez et al., 2005; Mazzoni et al., 2005; Bisson and Torre, 2011). During swimming the AFR of several motoneurons was very similar to the AFR exhibited during pseudo-swimming, although some of them exhibited differences. During swimming, motoneurons 3 oscillated at high AFR (10 Hz), while in the pseudo–swimming cells 3, 102, and 109 had oscillations with a lower rate between 1 and 3 Hz (Figure 7B). The firing of motoneurons CV, L, and AE was below 0.5 Hz.

A hierarchical analysis was applied with the correlations of motoneurons identified by the AP sorting (Figures 7C,D). We excluded from the analysis the AE cells but we included in the analysis the inhibitory motoneurons 101 to compare the activities of pure contractors and elongators with their corresponding inhibitors. This analysis showed that motoneurons 3 were similarly correlated during both behaviors and were situated in the left arm of the dendrograms but with a different level of correlation (Figures 7C,D). The inhibitory motoneurons 119 changed their position in the dendrogram from the right arm during swimming (demonstrating total anti-correlation with cell 3) to the left arm during pseudo-swimming (Figure 7D). Motoneurons CV and L showed correlated activity and were grouped together, but each type was located centrally and in opposite arms of dendrograms (central part Figures 7C and D). Motoneurons 102, were grouped during swimming, nevertheless, in pseudo-swimming, they were dispersed over the dendrogram. Motoneurons 109 were dispersed in the right arm of the dendrogram (Figure 7D).

### Recovery of the behaviors from dendrograms

Dendrograms described in previous sections were obtained by analyzing the electrical activity during episodes of the same behavior, i.e., crawling, swimming and pseudo-swimming. We asked if these dendrograms could be used to identify a specific behavior and then to check the similarity of dendrograms obtained from different preparations.

To verify whether these dendrograms could be used to identify episodes of specific behaviors, within the same experiment we identified the dendrogram of a specific behavior such as D_crawling_, D_swimming_, and D_pseudo−swimming_ and we computed in windows of 1 s the dendrogram D of the measured electrical activity. In order to compare and establish whether D is similar to D_crawling_, D_swimming_, and D_pseudo−swimming_ we needed a similarity indexes (*D*′, *D*″) between two dendrograms *D*′ and *D*″. There are several ways to compare labeled trees or hierarchical clustering: Fowlkes (Fowlkes and Mallows, 1983) proposed to use as a measure of similarity, *B*_*k*_, derived from the matching matrix [*m*_*ij*_], formed by cutting the two labeled trees and counting the number of matching entries in the resulting *k* clusters in each tree. Wagner (Wagner and Wagner, 2007) produced a survey of the most important similarity measures that can be employed in comparing two clustering, and all can be adapted to the framework of hierarchical clustering, provided that cutting on both trees is performed. After testing the majority of these similarity measures between dendrograms—which are labeled trees—, we chose the Fowlkes-Mallows Index and the Mutual Information Index which provided very similar results.

**Figure 8:**
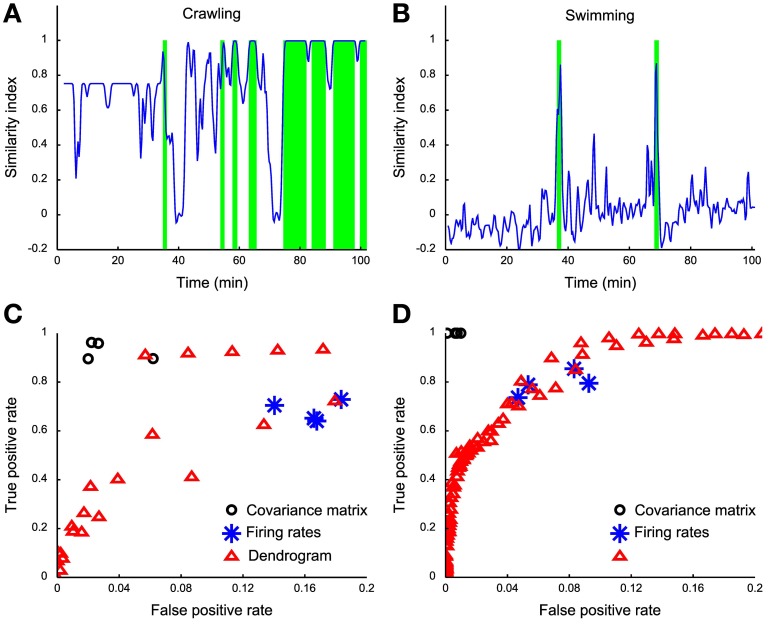
**Behavior detection using dendrograms. (A)** Time course of the similarity index of dendrograms estimated in a sliding window (1 s wide) using the crawling template dendrogram. **(B)** Time course of the similarity index of dendrograms estimated in a sliding window of 1 s using the swimming template dendrogram. **(C)** ROC analysis on different crawling classifiers: black dots represent covariance matrix classifiers (linear, diaglinear, quadratic, and diagquadratic, see Methods), blue asterisks represent firing rate classifiers (linear, diaglinear, quadratic, and diagquadratic, see Methods) and red triangles represent dendrogram classifiers based on mutual information similarity index with varying threshold *k* (see Methods). **(D)** Same as **(C)**, but for swimming.

Having established a reliable similarity index between the dendrogram *D* obtained from electrical recordings and the reference dendrograms *D*_behavior_ (*D*_crawling_, *D*_swimming_, and *D*_pseudo-swimming_) we investigated whether it was possible to identify crawling, swimming and pseudo-swimming by setting a threshold on s (*D*, *D*_behavior_). As shown in Figures 8A,B this procedure properly identified crawling and swimming in over more than 100 min of electrical recordings. Swimming episodes were rather easy to identify, as s (*D*, *D*_swimming_) was always below 0.5 with the exception of swimming episodes (Figure 8B). Although we successfully identified crawling and swimming, we could not recover pseudo-swimming episodes. A possible explanation for this failure is that our identification is based on electrical recordings obtained from mid-body ganglia. In pseudo-swimming the tail is attached to the bottom of the recording chamber and only the head moves, therefore electrical recordings from more rostral ganglia are expected to provide a better signature of this behavior.

Both behaviors could be more reliably detected by using a naïve classifier on the covariance matrix (Figures 8C,D, black circles), while a worse performance is obtained from the firing rates (Figures 8C,D, blue asterisks) compared to the dendrogram classifier (Figures 8C,D, red triangles). It is not surprising that the covariance classifier performs better than dendrograms because all the information contained in dendrograms is also contained in the covariance matrix. In our case the covariance matrix has more than 300 entries providing higher amounts of information, compared to the firing rate and dendrogram classifiers. The dendrogram classifier is an almost optimal compromise between reliable behavioral classification and simplicity and seems to capture the essence hidden in the covariance matrix. With these procedures we obtained only ~5% of false positives. For crawling, we obtain a better TPR (reaching 90% of correctly classified events) and ~5% of false positives. The low rate of false positives produced by the comparison of the two dendrograms is a clear index of the high specificity of each reference dendrogram, and thus of their reliability.

### Dendrograms from different experiments

If dendrograms were used to identify and characterize the electrical activity associated to specific behaviors, dendrograms associated to the same behavior, such as crawling in different animals *D*′_crawling_ and *D*″_crawling_ should be similar and in particular the distance between *D*′_crawling_ and *D*″_crawling_ should be smaller than the distance between the dendrograms of other behaviors from different preparations. Therefore, we attempted to identify average dendrograms, where the average was taken over different individuals, for the three most common behaviors: crawling, swimming and pseudo-swimming.

**Figure 9:**
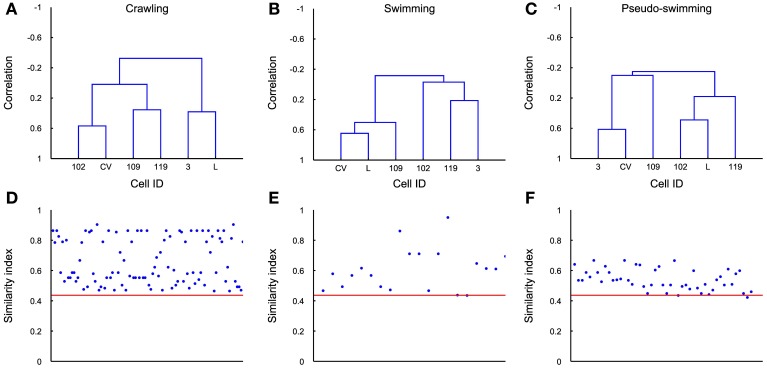
**Average dendrograms. (A–C)** The average dendrogram for crawling, swimming and pseudo-swimming, respectively, was estimated using all the recorded episodes in 5 experiments (for each of the behaviors) and using the average firing rates of the 6 most reliably identified cells. **(D–F)** Similarity index between the estimated dendrogram in each behavioral episode (blue dots) and the average dendrogram. Red line indicates the average over similarity indexes between all average dendrogram couples. Its low value represents good distinction among all behaviors.

The covariance matrix and the associated dendrogram recovered from different experiments could have a different number of entries, because the number of identified motoneurons varies in different experiments. If covariance matrices have different entries the associated dendrograms will be trees with a different number of leaves. In order to compare dendrograms from different experiments we averaged the firing activity of all motoneurons with the same identification, either at the left or right part of the ganglion, and located it either in the rostral or caudal ganglion. We also considered only 6 kinds of motoneurons, i.e., 3, L, CV, 102,109, and 119. From these dendrograms we obtained the average dendrogram (see Methods) associated to crawling <*D*_crawling_>, swimming <*D*_swimming_>, and pseudo-swimming <*D*_pseudo-swimming_>, which are reproduced in Figures 9A–C. For each of the behaviors we computed the similarity index between the average dendrogram and individual dendrograms of the same behavior, i.e., in the case of crawling s(*D*′_crawling_<D_crawling_>). This similarity index is reported in Figures 9D–F for all the behavioral episodes (blue dots) and a red horizontal line for the three behaviors. We then computed the distance between the average dendrogram of a specific behavior and all dendrograms of the other behaviors (blue dots in Figures 9D–F): this similarity indexes were consistently higher than the average over similarity indexes between all average dendrogram couples (red line). Therefore, by using an appropriate threshold, dendrograms can be used to characterize and identify stereotyped behaviors also in different animals.

## Discussion

In the present manuscript we have analyzed the simultaneous firing of some dozens of motoneurons and neurons during different behaviors, with the aim to identify patterns of the electrical activity characterizing specific behaviors in the leech. During crawling, swimming and pseudo-swimming specific patterns of correlation and anti-correlation among motoneurons emerge, which we describe using a dendrogram (Ward, 1963; Fowlkes and Mallows, 1983; Wagner and Wagner, 2007).

Dendrograms have been often used in Neuroscience to describe dendritic trees and their morphology (Faber et al., 2001; Duan et al., 2003; Zaborszky et al., 2005; Romand et al., 2011). Because dendrograms represent a type of hierarchical clustering, they have also been used to describe patterns of gene expression in neurons and neuronal tissues (Diaz, 2006) and sequence relationships among proteins of the same family (Pasquale, 2004). However, very rarely dendrograms have been used to describe patterns of electrical activity (Malhotra and Daucé, 2011) and how the degree of correlated activity changes during different behaviors, as described in the present work. Let us now discuss in detail these issues.

### Intact vs. semi-intact leeches

In order to compare electrical activity and behavior we have used a semi-intact leech preparation (Kristan and Stent, 1976), where one or two ganglia were exposed so to allow electrical recordings. Under these experimental conditions, by using 8 suction pipettes, it was possible to record patterns of electrical activity from several tens of motoneurons. When leeches were gently dissected, they could move easily their head and tail and could exhibit—although pinned down to the experimental dish—some of their stereotyped behaviors: indeed they could rhythmically shorten and elongate their whole body with almost the same frequency observed during unrestrained crawling (see Figure 3). Similarly, semi-intact leeches could move their head and tail in the same manner as during swimming and pseudo-swimming (Figures 3, 7). The statistics of sucker attachment and detachment of intact/pinned and semi-intact/pinned leeches (Figures 3, 4) was rather similar, suggesting that the gentle surgery necessary to obtain a semi-intact leech does not alter the leech behavior and the patterns of the underlying electrical activity.

### Sucker attachment and detachment

Suckers attachment and detachment have been analyzed in relation to the generation of the crawling behavior (Baader and Kristan, 1995; Baader, 1997; Baader and Bächtold, 1997). These events are essential for a correct crawling and must therefore be synchronized with contraction and elongation (Figures 6, 7). A correct timing and synchronization with motoneuron activity could be achieved by command neurons sending an appropriate neural signal along the connective fibers linking the head and tail sucker. Evidence for the existence of command neurons, possibly underlying head sucker detachment are shown in Figure 5, but we have not been able to identify specific command neurons for sucker detachment/attachment.

### Stationary states and correlated activity

Leeches in wilderness and in the tanks—kept for some months—spend a significant period of their time, immobile, in a stationary state. Therefore, we addressed the issue to identify which patterns of electrical activity are associated to these stationary states. During stationary states, the overall electrical activity decreases and indeed both the average firing rate <AFR_i_> and the average cross-correlation <ρ_*ij*_> have small values. However, as shown in Figure 2B, a small value of <ρ_*ij*_> is a better classifier than a small value of <AFR_i_> of stationary states. When the leech is not in a stationary state a change of <AFR_i_> is a better reporter than of <ρ_*ij*_> of changes of the tail or head velocity (Figure 3).

Global changes of the degree of correlated activity are also relevant in brain areas of higher vertebrates, where they are associated to specific cognitive functions. Changes of the degree of correlated electrical activity have been associated to attention (Goldberg et al., 2002; Ghosh et al., 2009; Moreno-Bote and Parga, 2010; Pesaran, 2010; Putrino et al., 2010). A higher correlated electrical activity seems to be essential for memory formation and consolidation. Modulations in the gamma-frequency and theta-frequency (4–8 Hz) ranges have been proposed to underlie memory performance and in particular to AP timing-dependent plasticity (Jutras and Buffalo, 2010). As a consequence, electrical synchronization is essential for memory formation underlying the cellular mechanisms of memory storage in higher vertebrates. In leeches, absence of correlated firing seems primarily related to a stationary state in which the animal is at rest and does appear to be associated to memory formation.

### Dendrograms and leech behavior

Covariance-based dendrograms provide a functional grouping of motoneurons with similar patterns of electrical activity. Dendrograms show clearly that during the contraction phase of crawling several longitudinal excitatory motoneurons, such as motoneuron 3 and L, increase their AFR whereas other motoneurons, such as the AE, CV, 102, 109 decrease their AFR, and during the elongation this firing pattern is reversed. They reveal also new properties of the reorganization of motoneuron firing during different behaviors. Dendrograms of contractions and elongations during different crawling cycles are similar, but contractions and elongations during crawling have a different structure during generic contractions and elongations. This is clearly visible by comparing those obtained during crawling (Figure 5) and generic contractions/elongations (Figure 6). Firing of motoneurons L and 3 are more correlated during crawling than during generic contractions/elongations.

Dendrograms also show more subtle features of the functional relationship established between the same motoneurons in two adjacent ganglia or in the left or right hemi ganglion according to the specific behavior and the type of motoneurons (Figure 5D). The electrical activity of the two longitudinal motoneurons 3 and L in neighboring ganglia and in the left and right hemi ganglion have a cross-correlation larger than 0.6 during crawling. Other motoneurons, such the two flattener motoneurons CV and 109 do not have a similar coordinated firing. The grouping of motoneurons with similar patterns of electrical activity is in agreement with the electrophysiological investigations of different leech behaviors (Friesen, 1989; Baader and Kristan, 1995; Brodfuehrer et al., 1995; Baader, 1997; Kristan et al., 2005; Briggman and Kristan, 2006; Briggman et al., 2006; Friesen and Kristan, 2007).

### Dendrograms and the time scale of leech behaviors

**Figure 10:**
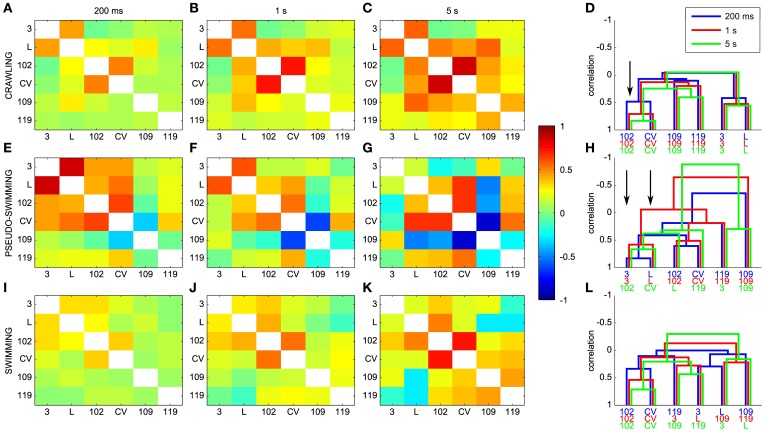
**Dendrograms and the time scale of leech behaviors. (A–C)** Dependence of the cross-covariance matrices on the window ΔT used for computing cross-covariances for crawling. ΔT was 200 ms, 1 s and 5 s in **(A**–**C**), respectively. **(E–G)** Same as in **(A–C)** but for pseudo-swimming. **(I–K)** Same as in **(A–C)**, but for swimming. **(D,H,L)** Dependence of dendrograms on ΔT for crawling, pseudo-swimming, and swimming, respectively. Dendrograms obtained with values of ΔT of 200 ms, 1 and 5 s in blue, red and green, respectively. Black arrows indicate the increase in the degree of correlated activity (see text).

As described in the Methods, dendrograms are obtained from the cross-covariance matrices ρ_*jk*_(ΔT) and represent a graphical way to capture the pattern of the electrical activity during different behaviors. A comparison between ρ_*jk*_(ΔT) and dendrograms is shown in Figure 10 for different behaviors and values of the window ΔT used for computing cross-covariance. For crawling, the structure of dendrograms does not change when ΔT is varied between 200 ms and 5 s (Figure 10D). In contrast, both for swimming and pseudo-swimming, the structure of dendrograms changes significantly when ΔT is increased from 200 ms to 5 s (see Figures 10H,L). During crawling, when ΔT is increased from 200 ms to 5 s, the degree of correlated activity among pairs of motoneurons with similar properties, such as (L and 3), (CV and 102) and (109 and 110) increases (arrow in Figure 10D) but the shape of dendrograms is not altered. Indeed, crawling occurs on a time scale of 5–10 s during which motoneurons do not change their relative firing. For swimming, which has a periodicity of around 1 s (see Figure 7), dendrograms do not change when ΔT is increased from 200 to 500 ms, but those dendrograms computed with values of Δ equal to 5 s have a completely different structure (Figure 10L): in fact motoneurons L and 109 which show a clear correlation with values of ΔT between 200 and 500 ms become anti-correlated when ΔT is equal to 5 s. During pseudo-swimming, dendrograms change their structure also when ΔT is increased from 200 to 500 ms and the correlation between motoneurons CV and 102 is lost (arrows in Figure 10H).

### Dendrogram robustness

Dendrograms, as other tools of data analysis, must be carefully evaluated and their robustness to noise and its sensitivity to used parameters must be examined. We verified that dendrograms (obtained for 5 experiments) do not change their structure when the boundary points of used intervals are shifted by ΔT/2. As shown in Figure 10, however, dendrograms change their structure when the bin size ΔT is varied. In order to circumvent this problem, it is possible to use a binless method to obtain firing rates (Victor, 2002; Masud and Borisyuk, 2011), but neurons in the leech nervous system and in all known nervous systems have characteristic firing rate which governs their functional properties: indeed the comparison of dendrograms obtained for different values of ΔT (Figure 10) reveals the characteristic time of each behavior.

We have shuffled the data shown in Figure 5 by shifting the electrical recordings, procedure reminiscent of frame shifting of Beggs and Plenz (2004). If electrical recordings from the DP (or AM) nerve are shifted by less than 5 s the structure of dendrograms did not change, but changed when time shifts were larger than 10 s. When data obtained from the DP and AM nerves were mixed, because of shuffling and/or bootstrapping, dendrograms completely changed, as expected from the mixing of the electrical activity of motoneurons with well-defined different properties, underlying elongation and contraction.

Data of Figure 9 represent a good test of the robustness of dendrograms as a tool to relate electrical activity to behavior. Indeed dendrograms associated to crawling or other behaviors in different animals should be similar. As shown in Figure 9 the similarity index between the same behavior in different animals is almost always larger than the similarity index between different behaviors. This observation indicates that dendrograms are robust against the variability of biological preparations and of the intrinsic experimental variability.

### Dendrograms as a general tool for understanding reconfigurable neuronal networks

Dendrograms, here used, are based on the analysis of the matrix of cross-covariance among identified motoneurons and represent a compact description of the information embedded in neural structures: ganglia. Our approach is reminiscent of a similar analysis of pattern of electrical activity in cat auditory cortex (Eggermont, 2006) and rat Pontine Parabrachial Nuclei (Nishijo and Norgren, 1991).

Dendrograms were estimated using agglomerative algorithms because they are computationally less demanding than others. Agglomerative algorithms start with *n* singleton classes and at each stage, the most similar pair of classes is amalgamated: they can be divided into single linkage (shortest distance), complete linkage (furthest distance) and incremental sum of squares (Ward, 1963). Some of these methods (e.g., Ward's linkage) require objects to be represented by points in some Euclidean space, and the measure of pairwise dissimilarity to be proportional to a squared Euclidean distance. Johnson (1967) has shown that single linkage is a connectivity method favoring elongated clusters of objects, while complete linkage provides more compact clusters with a minimum diameter. The single linkage method has been used in biological sciences, where clustering specimens that are minimally different is felt to correspond to the pattern of evolutionary changes (Cole and Wishart, 1970). The complete linkage method is used extensively in social sciences where compact clusters and internally homogeneous are desirable (Baker, 1974).

Dendrograms provide a good mathematical tool to decipher and describe how neurons and motoneurons change their orchestrated activity according to specific behaviors and tasks and their use is expected to be fruitful not only in the leech nervous system but in all reconfigurable neuronal networks when the aim is to understand the underlying changes of correlation patterns.

### Conflict of interest statement

The authors declare that the research was conducted in the absence of any commercial or financial relationships that could be construed as a potential conflict of interest.
